# The efficacy of addition of Tenofovir Disoproxil Fumarate to Peg-IFNα-2b is superior to the addition of Entecavir in HBeAg positive CHB patients with a poor response after 12 weeks of Peg-IFNα-2b treatment alone

**DOI:** 10.7150/ijms.45658

**Published:** 2020-06-08

**Authors:** Sheng Lin, Ya Fu, Wennan Wu, Tianbin Chen, Ningdai Chen, Zhen Xun, Can Liu, Qishui Ou, Yongbin Zeng, Huanhuan Huang

**Affiliations:** 1 Department of Laboratory Medicine, Fujian Key Laboratory of Laboratory Medicine, The First Affiliated Hospital of Fujian Medical University, Fujian, China.; 2 Department of Pediatrics, The First Affiliated Hospital of Fujian Medical University, Fujian, China.

**Keywords:** Chronic Hepatitis B, Tenofovir, Entecavir, Interferon-α, Combination Therapy

## Abstract

**Background:** There are limited data regarding the efficacy of addition of entecavir (ETV) or tenofovir disoproxil fumarate (TDF) to Peg-IFNα-2b in HBeAg positive chronic hepatitis B (CHB) patients without early response to Peg-IFNα-2b. In this study, we aimed to evaluate the efficacy of ETV and TDF in HBeAg positive CHB patients who had a poor response to Peg-INFα-2b at the end of 12 weeks of monotherapy.

**Methods:** A total of 40 HBeAg-positive CHB patients who were naive to antiviral therapy were recruited. The patients received a subcutaneous injection of Peg-IFNα-2b (180 µg) once a week for 12 weeks. However, the patients had a poor response to Peg-INFα-2b at the end of the 12-week-period monotherapy. The patients were then divided into two therapeutic protocol groups: (1) Group A: Patients received Peg-IFNα-2b (180 µg) subcutaneously weekly and ETV (0.5 mg) orally once daily for 48 weeks; (2) Group B: Patients received Peg-IFNα-2b (180 µg) subcutaneously weekly and TDF (300 mg) orally once daily for 48 weeks. The therapeutic efficacy was evaluated. Blood samples were collected at baseline and every 12 weeks. Routine biochemical tests including ALT, AST, etc. were measured by automated biochemical technique. HBV DNA was quantified using the TaqMan PCR assay. The levels of HBsAg, HBsAb, HBeAg, HBeAb and HBcAb were measured using a commercial chemiluminescent microparticle immunoassay.

**Results:** The HBsAg level declined rapidly in both two treatment groups during the first 12 weeks and declined gradually in the next 36 weeks. At week 48, the mean ΔHBsAg level in Peg-IFNα-2b+TDF group was significantly higher than that in Peg-IFNα-2b +ETV group (-1.799 ± 0.3063 vs. -1.078 ± 0.2028, *P*=0.0491). The HBeAg loss rate was significantly higher in TDF add-on group than that in ETV add-on group at week 48 (40% vs. 10%, *P*=0.028). At week 48, the proportions of patients with undetectable HBV DNA (<500 IU/mL) were 80% (16 out of 20) and 95% (19 out of 20) in Peg-IFNα-2b+ETV group and Peg-IFNα-2b+TDF group, respectively.

**Conclusions:** This real world study demonstrated that the efficacy of addition of TDF to Peg-IFNα-2b is superior to the efficacy of addition of ETV to Peg-IFNα-2b in HBeAg positive CHB patients with a poor response after 12 weeks of Peg-IFNα-2b treatment alone. However, this present study also requires a larger sample size study to verify in the future.

## Introduction

Hepatitis B virus (HBV) infects approximately 250 million people globally and is an important cause of liver disease covering chronic hepatitis, liver cirrhosis and hepatocellular carcinoma (HCC)^1^. At present, nucleos(t)ide analogues (NAs) [such as entecavir (ETV), tenofovir disoproxil fumarate (TDF) and tenofovir alafenamide (TAF)] and interferon-α (IFNα), are used for the treatment of chronic hepatitis B (CHB) 2. However, due to the presence of covalently closed circular DNA (cccDNA) in hepatocytes, it is hard to fully eliminate HBV from infected hepatocytes 3.

NAs can effectively inhibit HBV replication by blocking the process of reverse transcription 4. In contrast, IFNα possesses both antiviral and immunomodulatory functions, and may accelerate degradation of cccDNA, decrease its transcription 5, 6. However, the two types of antiviral agents have their advantages and disadvantages. NAs are effective inhibitors of HBV replication and suitable for long-term treatment 4. However, virological recurrence develops frequently after withdrawal of NAs treatment in patients with CHB who have achieved seroconversion and completed additional consolidation therapy. In an review, Seng Gee Lim documented NAs treatment withdrawal led to relapse in 50% of patients who achieved HBeAg seroconversion and completed at least 12 months of consolidation therapy 7. Though IFNα is more efficacious than NAs in the treatment of CHB patients, only about 30% of patients will respond, and the therapy with IFNα is less tolerated and cause more side effects 8, 9. For the different mechanisms of the two agents and before the new drugs emerge, many studies had focus on investigating effective regimens aiming to improve the overall efficacy of pegylated IFN-α in clinical practice 10-12. For example, Yan *et al*. documented that addition of adefovir dipivoxil (ADV) or ETV to Peg-IFNα-2a enhanced virological response in CHB patients without early response to Peg-IFNα-2a 10. However, no significant difference was observed between combination therapy and monotherapy in other research 13, 14.

To date, there are still limited data regarding the efficacy of addition of ETV or TDF to Peg-IFNα-2b in HBeAg positive CHB patients without early response to Peg-IFNα-2b. Consequently, in this study, we evaluated the efficacy of addition of ETV and TDF in HBeAg positive CHB patients who had a poor response to Peg-INFα-2b at the end of 12 weeks of monotherapy.

## Methods

### Patient recruitment

A total of 40 HBeAg-positive CHB patients who were naive to antiviral therapy were recruited in the Center of Liver diseases of the First Affiliated Hospital of Fujian Medical University from June 2017 to October 2017. The CHB diagnosis was conformed according to Guideline of Prevention and Treatment for Chronic Hepatitis B (2015 Version), enacted by the Chinese Society of Hepatology and Chinese Society of Infectious Diseases, Chinese Medical Association. The study was approved by the ethics committee of The First Affiliated Hospital of Fujian Medical University. All patients signed an informed consent form for this study. Blood samples were collected at baseline and every 12 weeks (week 0, week 12, week 24, week 36 and week 48).

The inclusion criteria were as follows: (1) HBsAg positive for at least 6 months; (2) HBeAg positive; (3) persistent level of HBV DNA ≥20 000 IU/mL; and (4) ALT ≥2 and ≤10 × upper limit of normal. Exclusion criteria included: Patients treated with other antiviral drugs, co-infected with HCV, HIV, or HDV, cirrhosis, hepatocellular carcinoma (HCC), autoimmune hepatitis, association with other severe underlying disease. Patients with incomplete course of therapy or follow-up were excluded as well.

### Therapeutic protocol

The enrolled patients received a subcutaneous injection of Peg-IFNα-2b (180 μg) once a week for 12 weeks; However, the patients had a poor response to Peg-INFα-2b at the end of the 12-week-period monotherapy. The patients were then divided into two therapeutic protocol groups after communication with patients and getting their agreements: (1) Group A: Patients received Peg-IFNα-2b (180 μg) subcutaneously weekly and ETV (0.5 mg) orally once daily for 48 weeks; (2) Group B: Patients received Peg-IFNα-2b (180 μg) subcutaneously weekly and TDF (300 mg) orally once daily for 48 weeks.

### Laboratory measurements

Blood samples were collected at every visit time. Routine blood testing was performed on Siemens ADVIA 2120 Hematology Analyzer. Routine biochemical tests including alanine aminotransferase (ALT), aspartate aminotransferase (AST), gamma-glutamyltransferase (GGT), etc. were measured by automated biochemical technique (Siemens Healthcare Diagnostics, USA). Serum HBV DNA was quantified using the TaqMan polymerase chain reaction (PCR) assay (Sansure Biotech, China) on ABI7500 Real‐Time PCR System (Life Technologies, USA), which has a detection limit of 500 IU/mL. The levels of HBsAg, HBsAb, HBeAg, HBeAb and HBcAb were measured using a commercial chemiluminescent microparticle immunoassay kit with the Architect i4000SR System (Abbott Laboratories, USA).

### Determination of therapeutic efficacy

Therapeutic efficacy assessments were conducted every 12 weeks. Biochemical response was defined as normalization of ALT (ALT ≤40 IU/L), serologic response was defined as loss of HBeAg with or without HBeAg seroconversion, and virologic response was defined as reduction of HBV-DNA level to <500 IU/mL.

### Statistical Analysis

HBV DNA and HBsAg concentration was log10 transformed for analysis. The statistical analysis and graphing were performed using statistical analysis software SPSS version 23.0 (SPSS Inc, USA) and GraphPad Prism software version 6.0 (GraphPad Software, USA).One-sample Kolmogorov-Smirnov test was used to determine the distribution of each group data. Data following a normal distribution was expressed as mean ± SD. Continuous variables were conducted using the Student's *t*-test. The categorical variables were analyzed using the χ^2^ test. *P*<0.05 was considered as statistical significance.

## Results

### Baseline characteristics of HBeAg-positive CHB patients receiving different therapeutic protocols in this study

The baseline characteristics of HBeAg-positive CHB patients were described in **Table 1**.

### On-treatment HBsAg decline

**Figure 1:**
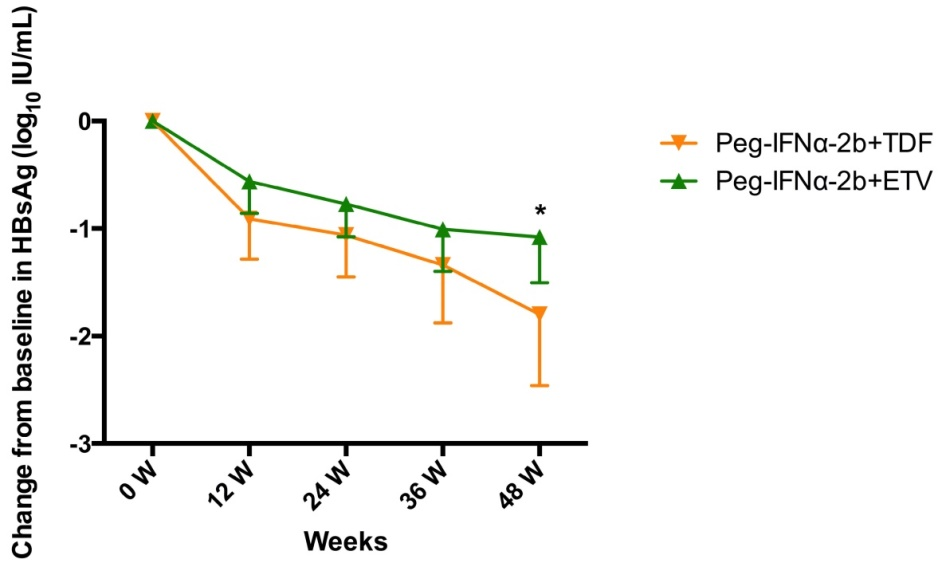
Change in HBsAg level (log_10_ IU/mL) from baseline to week 48 stratified by different therapeutic protocols. Data shown are mean±95% confidence intervals.

As shown in **Figure 1**, the HBsAg level declined rapidly in the treatment groups during the first 12 weeks and declined gradually in the next 36 weeks. The mean HBsAg levels reductions (ΔHBsAg±SD) were 0.73±0.60, 0.28±0.29, 0.56±0.63, 0.91±0.67 at week 12, 24 and 36, respectively. At week 48, the mean ΔHBsAg level in Peg-IFNα-2b+TDF group was significantly higher than that in Peg-IFNα-2b+ETV group (-1.799 ± 0.3063 vs. -1.078 ± 0.2028, *P*=0.0491).

### HBeAg loss

**Figure 2:**
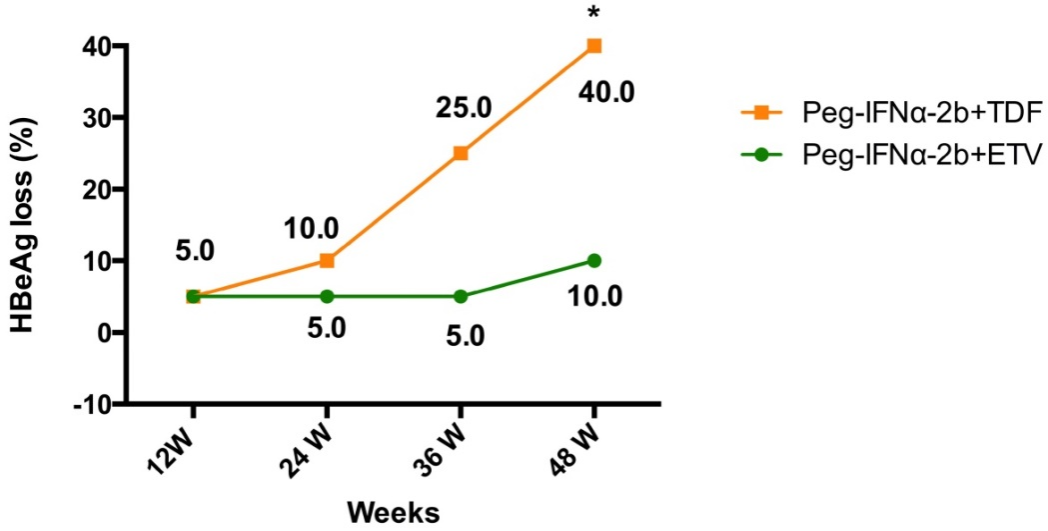
Evolution of HBeAg loss in patients with HBeAg positive chronic hepatitis B treated with Peg-IFNα-2b+ETV or Peg-IFNα-2b+TDF.

At week 48, the percentages of HBeAg loss were 10% (2 out of 20) in patients assigned to the ETV add-on group, and 40% (8 out of 20) in patients assigned to the TDF add-on group (**Table 2** and **Figure 2**). The HBeAg loss rate was significantly higher in TDF add-on group than that in ETV add-on group at week 48 (40% vs. 10%, *P*=0.028). From week 24 to 36, 6 additional patients in the TDF add-on group compared to 1 in the ETV add-on group had HBeAg loss.

### Changes in HBV DNA

At week 48, the proportions of patients with undetectable HBV DNA (<500 IU/mL) were 80% (16 out of 20) and 95% (19 out of 20) in Peg-IFNα-2b+ETV group and Peg-IFNα-2b+TDF group, respectively (**Table 2**).

### ALT normalization and biochemical response

At week 48, 14 out of 20 patients (70%) in Peg-IFNα-2b+ETV group had normal ALT (ALT ≤ 40 IU/L), 11 out of 20 patients (55%) in Peg-IFNα-2b+TDF group had normal ALT. However, differences in the proportions of ALT normalization were not statistically significant. The rate of normalization of ALT comparable at week 12, 36 and 48 in the two different therapeutic protocols groups were shown in **Table 2.**

### Dynamic changes in lymphocyte, neutrophil, erythrocyte and red blood cell distribution width

**Figure 3:**
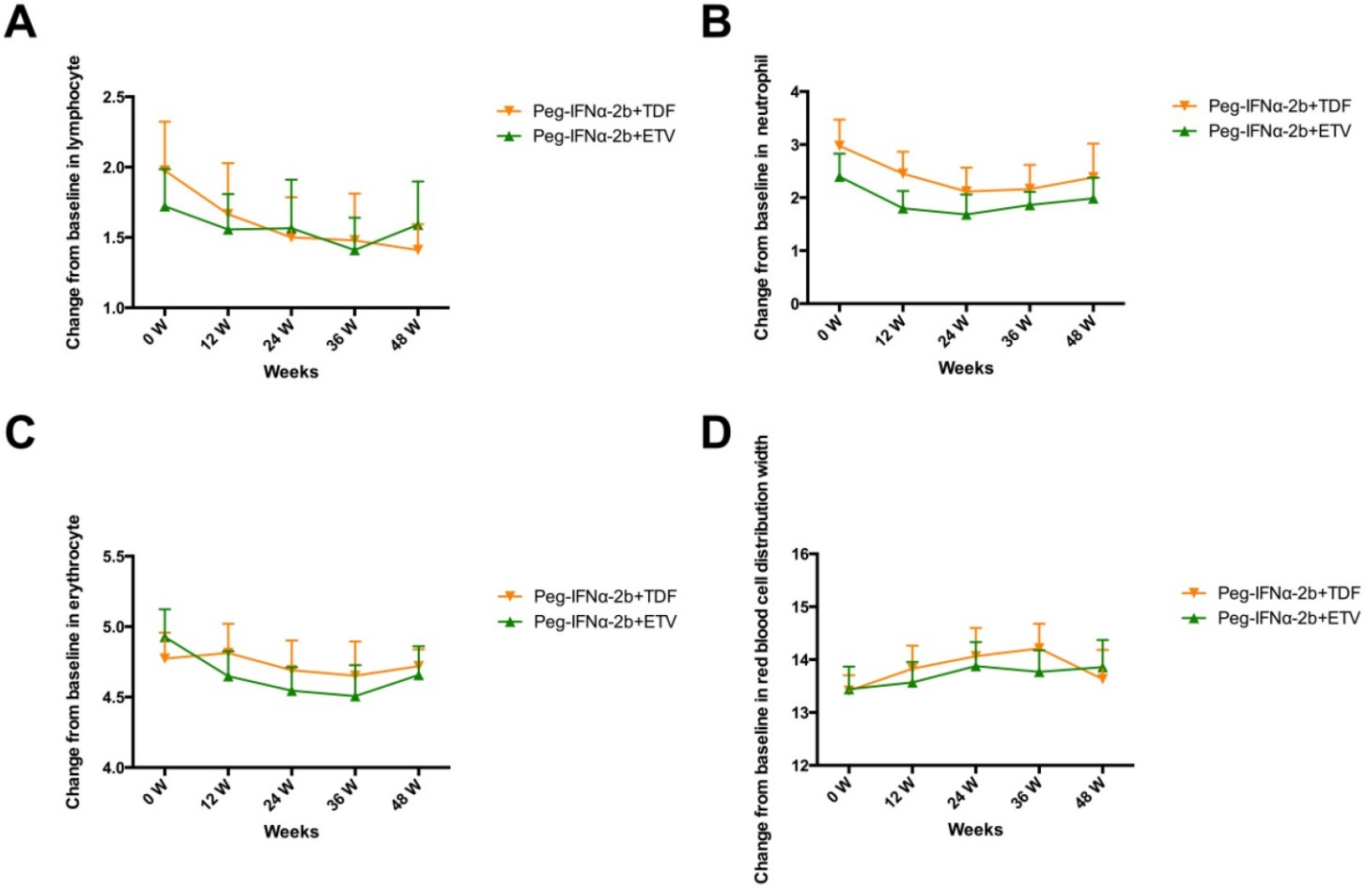
Dynamic changes in lymphocyte (A), neutrophil (B), erythrocyte (C) and red blood cell distribution width (D).

As shown in **Figure 3**, the patients of the two groups all experienced decreases in lymphocytes, neutrophils and erythrocyte, however, the differences did not reach statistical significance. The red blood cell distribution width (RDW) slightly increased in the two groups.

## Discussion

Owing to different mechanisms of the IFNα and NAs, increasing studies have conducted sequential NAs to Peg-IFNα strategy or de novo Peg-IFNα + NAs combination for treatment of CHB. Currently, attention had been paid to the combination therapy with Peg-IFNα and ETV/TDF (ETV and TDF were recommended as first-line treatment NAs); and suggested that Peg-IFNα combined with TDF or ETV can further improve the response rate of CHB patients. In an open-label, active-controlled study, Patrick Marcellin *et al* found, at week seventy-two of follow-up, the rate of HBsAg loss was significantly higher in patients receiving TDF plus peginterferon for 48 weeks than in those receiving monotherapy with either TDF or Peg-IFNα 12. Li* et al* documented IFNα plus ETV therapy can accelerate HBsAg decline as compared with IFNα monotherapy in CHB patients with lower baseline HBsAg levels 11. However, there were still few reports on the efficacy of addition of ETV or TDF to Peg-IFNα-2b in HBeAg positive CHB patients with a poor response after 12 weeks of Peg-IFNα-2b treatment alone.

In the present study, both the two regimens of combination therapy led to a significantly greater HBV DNA levels decline over time. At week 48, the proportion of patients with undetectable HBV DNA in Peg-IFNα-2b+TDF group was higher than that in Peg-IFNα-2b+ETV group (95% VS 80%), however, no significant difference was observed. The rate of ALT normalization was comparable at weeks 12, 24, 36 and 48 in both treatment groups. On-treatment HBsAg levels can be helpful for the prediction of a treatment response for CHB patients. Janssen HL *et al* found that patients with HBsAg levels >20000 IU/mL at week 24 have a low probability of response, irrespective of HBV genotype; while patients with HBsAg levels <1500 IU/mL at week 12 had a high probability of response^15^. Several studies have documented patients receiving Peg-IFNα monotherapy exhibited significantly lower reduction in HBsAg levels compared to patients receiving combination therapy with NAs and Peg-IFNα^11^, ^16^-^18^. In the present study, CHB patients in the Peg-IFNα-2b+TDF group had a more significant change in serum HBsAg levels compared to CHB patients in the Peg-IFNα-2b+ETV group after 48 weeks treatment. HBeAg loss and the appearance of anti-HBe (HBeAg seroconversion) is believed to be a low risk of disease progression 19. Moreover, patients with HBeAg seroconversion have a reduced risk of hepatocellular carcinoma and cirrhosis; and patients who do not achieve HBeAg seroconversion are more likely to rebound HBV DNA after discontinuation 20. Therefore, HBeAg seroconversion is an important therapeutic endpoint for the management of HBeAg-positive CHB and is often evaluated as a biomarker during Peg-IFNα therapy. While in this study, only 10 of the 40 patients (25%) achieved HBeAg loss. The HBeAg seroconversion rate was low because the study performed only after the end of treatment and the insufficient following-up data available. Consequently, a long time is required to observed HBeAg loss and seroconversion. On adverse effects, neutrophil, lymphocyte and erythrocyte declined slightly after Peg-IFNα-2b+ETV or Peg-IFNα-2b+TDF treatment. Both the two regimens had little influence on red blood cell count and red blood cell distribution width.

In summary, this real world study demonstrated that the efficacy of addition of TDF to Peg-IFNα-2b was superior to the addition of ETV in HBeAg positive CHB patients with a poor response after 12 weeks of Peg-IFNα-2b treatment alone. Peg-IFNα-2b+TDF therapy was superior to Peg-IFNα-2b+ETV therapy in increasing the proportion of HBeAg loss and the decline of HBsAg level. This present study also requires a larger sample size study to verify in the future. Moreover, long-term and off-treatment follow-up are needed to investigate the issue of sustained antiviral effects.

## Figures and Tables

**Table 1:** The baseline characteristics of HBeAg‐positive CHB patients in the two different therapeutic protocols groups

Characteristic	Peg-IFNα-2b+ETV	Peg-IFNα-2b+TDF	Statistical value	*P* value
Number	20	20	NA	NA
Age, years	28.75±4.833	29.94±5.157	-0.682	0.500
Gender, n Male/n Female	15/5	17/3	2.500	0.134
HBsAg, log_10_ IU/mL	4.0173±0.81801	4.2604±0.63169	-1.235	0.225
HBeAg, log_10_ S/CO	2.607±0.7371	2.809±0.81298	-1.454	0.156
ALT, U/L	187.95±150.906	143.69±115.446	1.008	0.321
AST, U/L	100.15±96.808	77.94±52.372	0.914	0.367
GGT, U/L	56.2±34.935	76.38±81.127	-0.847	0.407
RDW	13.69±1.16	13.388±0.5830	1.037	0.308
Erythrocyte, mean ± SD (10^12^/L)	4.857±0.40181	4.8325±0.34265	0.343	0.445
Lymphocyte, mean ± SD (10^12^/L)	1.722±0.56253	1.9150±0.64759	-1.018	0.316
Neutrophil, mean ± SD (10^12^/L)	2.396±0.92125	3.1831±1.40737	-1.096	0.055
HBV DNA, log_10_ IU/mL	6.9205±1.81974	7.426±0.99468	-1.092	0.283

Continuous variables were expressed as means ± standard deviation. ALT: alanine aminotransferase, AST: aspartate aminotransferase, GGT: gamma-glutamyltransferase, RDW: red blood cell distribution width, NA: not applicable.

**Table 2:** Rate of HBeAg loss, ALT normalization and undetectable HBV DNA

Week	Peg-IFNα-2b+ETV (n=20), n (%)	Peg-IFNα-2b+TDF (n=20), n (%)	*P* value
HBeAg loss			
Week 12	1(5.0)	1 (5.0)	1.000
Week 24	1 (5.0)	2 (10.0)	1.000
Week 36	1 (5.0)	4 (25.0)	0.342
Week 48	2 (10.0)	8 (40.0)	0.028
ALT normalization			
Week 12	6 (30.0)	4 (20.0)	0.465
Week 24	6 (30.0)	6 (30.0)	1.000
Week 36	14 (70.0)	10 (50.0)	0.197
Week 48	14 (70.0)	11 (55.0)	0.327
HBV DNA undetectable			
Week 12	7 (35.0)	10 (50.0)	0.337
Week 24	11 (55.0)	14 (70.0)	0.327
Week 36	14 (70.0)	16 (80.0)	0.465
Week 48	16 (80.0)	19 (95.0)	0.342
